# Trends and spatial distribution of neonatal sepsis, Uganda, 2016–2020

**DOI:** 10.1186/s12884-023-06037-y

**Published:** 2023-11-04

**Authors:** Stella M. Migamba, Esther Kisaakye, Allan Komakech, Miriam Nakanwagi, Petranilla Nakamya, Robert Mutumba, Deogratius Migadde, Benon Kwesiga, Lilian Bulage, Daniel Kadobera, Alex R. Ario

**Affiliations:** 1 Uganda Public Health Fellowship Program, Uganda National Institute of Public Health, Kampala, Uganda; 2 Reproductive and Infant Health Division, Ministry of Health, Kampala, Uganda

**Keywords:** Neonatal sepsis, Early-onset sepsis, Late-onset sepsis, Uganda

## Abstract

**Background:**

In Uganda, sepsis is the third-leading cause of neonatal deaths. Neonatal sepsis can be early-onset sepsis (EOS), which occurs ≤ 7 days postpartum and is usually vertically transmitted from the mother to newborn during the intrapartum period, or late-onset sepsis (LOS), occurring 8–28 days postpartum and largely acquired from the hospital environment or community. We described trends and spatial distribution of neonatal sepsis in Uganda, 2016–2020.

**Methods:**

We conducted a descriptive incidence study using routinely-reported surveillance data on in-patient neonatal sepsis from the District Health Information System version 2 (DHIS2) during 2016–2020. We calculated incidence of EOS, LOS, and total sepsis as cases per 1,000 live births (LB) at district (n = 136), regional (n = 4), and national levels, as well as total sepsis incidence by health facility level. We used logistic regression to evaluate national and regional trends and illustrated spatial distribution using choropleth maps.

**Results:**

During 2016–2020, 95,983 neonatal sepsis cases were reported, of which 71,262 (74%) were EOS. Overall neonatal sepsis incidence was 17.4/1,000 LB. EOS increased from 11.7 to 13.4 cases/1,000 LB with an average yearly increase of 3% (p < 0.001); LOS declined from 5.7 to 4.3 cases/1,000 LB with an average yearly decrease of 7% (p < 0.001). Incidence was highest at referral hospitals (68/1,000 LB) and lowest at Health Center IIs (1.3/1,000 LB). Regionally, total sepsis increased in Central (15.5 to 23.0/1,000 LB, p < 0.001) and Northern regions (15.3 to 22.2/1,000 LB, p < 0.001) but decreased in Western (23.7 to 17.0/ 1,000 LB, p < 0.001) and Eastern (15.0 to 8.9/1,000, p < 0.001) regions.

**Conclusion:**

The high and increasing incidence of EOS in Uganda suggests a major gap in sepsis prevention and quality of care for pregnant women. The heterogenous distribution of neonatal sepsis incidence requires root cause analysis by health authorities in regions with consistently high incidence. Strengthening prevention and treatment interventions in Central and Northern regions, and in the most affected districts, could reduce neonatal sepsis. Employment of strategies which increase uptake of safe newborn care practices and prevent neonatal sepsis, such as community health worker (CHW) home visits for mothers and newborns, could reduce incidence.

## Background

Neonatal sepsis is a clinical syndrome that includes pneumonia and meningitis. It is a severe bacterial infection in the first month of life. Early-onset sepsis (EOS) occurs in the first seven days of life and is caused by intrapartum transmission of bacteria from the mother to the neonate [1–3]. Late-onset sepsis (LOS) occurs from 8 to 28 days of life and is usually acquired postnatally from the hospital or community environment [1]. Both forms are characterised by a combination of temperature instability, difficulty breastfeeding, seizures, respiratory distress, jaundice, vomiting, diarrhoea, abdominal distention, or diminished activity [4].

Approximately 1.3 million cases of neonatal sepsis occur annually [5], and in 2018, 15% of neonatal deaths globally were due to sepsis [6]. In resource-rich settings with lower neonatal death rates, approximately 9–15% of neonatal deaths are due to sepsis, whereas in resource-limited settings like Africa with high neonatal death rates, sepsis accounts for 23–27% of neonatal deaths [7]. Risk factors for neonatal sepsis include prematurity, low birth weight (< 2.5 kg), premature rupture of membranes, prolonged labour, caesarean section delivery, maternal infection, and lack of antenatal care [5, 8–10]. Home births and lack of skilled birth attendants are also associated with increased risk of neonatal sepsis [11]. In 2016, 73% of deliveries in Uganda occurred in healthcare facilities; 74% of deliveries were attended by skilled birth attendants [11]. Diagnosis of neonatal sepsis in Uganda, like other low- and middle-income countries, often relies on a clinical diagnosis of sepsis based on WHO criteria instead of a blood culture as occurs in resource-rich settings [10, 12–15].

A recent study of neonatal sepsis among facility births in low- and middle-income countries in Asia and Africa found an incidence of 166/1,000 live births (LB) for clinically suspected sepsis, and 47/1,000 LB for laboratory (blood culture)-confirmed sepsis [16]. A systematic review and meta-analysis estimated the pooled neonatal sepsis incidence in the WHO African Region at 52.4 cases/1,000 LB during 1979–2019. Among four studies in low- and middle-income countries conducted during 2009–2018 that reported both clinically-diagnosed and (sometimes) laboratory-confirmed sepsis, the incidence was 39.3/1,000 LB [14].

In Uganda, neonatal sepsis is the third-leading contributor to neonatal mortality, behind birth asphyxia and preterm birth complications. The recommended first-line treatment for neonatal sepsis in Uganda is administration of intravenous ampicillin, with gentamycin and cephalosporins recommended as second-line drugs [15, 17]. Strategies have been implemented to attain the 2030 WHO target of ≤ 12 newborn deaths per 1,000 LB [18]. Despite the implementation of multiple interventions in Uganda during 2002–2016, including kangaroo mother care, breastfeeding within one hour after birth, umbilical cord care, use of tetracycline eye ointment for prevention of eye infections, and vitamin K administration [6, 11, 17, 19], the neonatal mortality rate (NMR) remained essentially unchanged at approximately 27/1,000 LB [11]. The Maternal Perinatal Death Surveillance and Response report for the fiscal year 2019/2020 attributed 12% of newborn deaths in Uganda to neonatal sepsis [20].

While there is some available literature on neonatal sepsis in Uganda [10, 21–23], studies describing the distribution of neonatal sepsis cases geographically and over time are rare. We analyzed the trends and spatial distribution of neonatal sepsis in Uganda during 2016–2020 to guide interventions to reduce the incidence of neonatal sepsis and sepsis-related deaths.

## Methods

### Study setting

Uganda’s projected population for 2022 was 43.7 million with an annual population growth rate of 3.1% [24]. As of 2016, the national fertility rate was 5.4 children per woman, one of the highest in the world, and the crude birth rate was 38.7 per 1,000 population [11]. Uganda has approximately 7,000 health facilities, of which 45% are government-owned, 40% are private-for-profit (PFP), 14% are private-not-for-profit (PNFP), and 1% are community-owned [25]. These health facilities are classified into seven levels: Clinic, Health Centre Two (HC II), Health Centre Three (HC III), Health Centre Four (HC IV), general hospital, regional referral hospital (RRH), and national referral hospital (NRH) (Table 1). Clinic facility services are community based preventive and promotive health services [25].

**Table 1:** Health facilities by level and region in Uganda

Region	Clinic	HC II	HC III	HC IV	Hospital	RRH	NRH	Total
Central	1,166	1,323	498	68	62	2	5	3,124
Eastern	161	694	380	52	37	3		1,327
Northern	118	554	320	33	29	4		1,058
Western	133	793	372	69	35	4		1,406
**Total**	**1,578**	**3,364**	**1,570**	**222**	**163**	**13**	**5**	**6,915**

The number, range, and complexity of services increase progressively with the level of the health facility. Maternity services and the recommended intravenous antibiotics for the management of neonatal sepsis are provided at HC III levels and above [25, 26]. The HC IV is the first referral facility that provides comprehensive obstetric and newborn care services in counties where there are no PNFP facilities [27]. Staffing level in public health facilities is at 74% with HC IIs, HC IIIs, HC IVs, and NRHs with 75% positions filled while RRHs have the lowest staffing at 69% [27]. There are approximately 1.9 health workers per 1,000 population compared to the WHO recommendation of 2.8 per 1,000 population needed to achieve universal health coverage [28].

### Study design, neonatal sepsis surveillance, and data source

We conducted a descriptive incidence study of routinely-reported, aggregated nationwide surveillance data on inpatient neonatal sepsis during 2016–2020 using the District Health Information System version 2 (DHIS2). DHIS2 is an electronic version of data from Uganda’s Health Management Information System (HMIS). The HMIS is a paper-based reporting system in which health facility data on several conditions, including neonatal sepsis, are reported on a weekly and monthly basis. In DHIS2, neonatal sepsis is categorised as inpatient or outpatient depending on whether the cases were reported in the ‘Health Unit Outpatient Monthly Report- HMIS Form 105’ or in the ‘Health Unit Inpatient Monthly Report- HMIS Form 108.’ Both inpatient and outpatient sepsis are further classified into early-onset (EOS; 0–7 days after birth) and late-onset (LOS; 8–28 days after birth) depending on time of presentation. Sepsis does not have to be lab-confirmed to be included in DHIS2. For the data extracted in this analysis, HMIS data flow from HC III to HC IV level facilities and then to the district. At the district, data from each health facility are entered by the district biostatistician into DHIS2. Regional and national referral hospitals send data directly to the Ministry of Health. At the Ministry of Health, data from all health facilities are collated. Data from 2016 to 2019 were obtained from a previous version of DHIS2 in which each health facility was required to submit just one inpatient monthly report that included all variables. In 2020, the DHIS2 was upgraded and currently requires submission of three inpatient reports per month per facility, including one report each for nationals, foreigners, and refugees.

### Study population

The study population comprised all records for infants aged 0–28 days (neonates) treated at health facilities at HC III level and above in Uganda during January 2016-December 2020.

### Study variables and data abstraction

For this analysis, we downloaded DHIS2 data elements on neonatal sepsis at 0–7 days of life, at 8–28 days of life, and total LB deliveries in a health unit. Only newborns captured as inpatient cases at health facilities were considered. We also downloaded 2016–2020 data from DHIS2 on the national health facility reporting rates for neonatal sepsis, defined as the proportion of facilities reporting among those expected to report, to identify possible impacts of fluctuations in reporting rates on apparent sepsis incidence; we obtained the combined reporting rate for HC IIIs, HC IVs, general hospitals, and regional and national referral hospitals. Data were exported from DHIS2 to Microsoft Excel and then into Epi Info 7 for analysis.

### Data analysis

We calculated incidence rates of EOS, LOS, and total (occurring at infant ages 0–28 days) neonatal sepsis during 2016–2020 at district, regional, and national levels. Incidence rates for total sepsis were also stratified by health facility level. Incidence rate was the number of new sepsis cases per 1,000 LB at health facilities. We reported regional and national trends in neonatal sepsis incidence and reporting rates using line graphs and determined the significance of changes in trends using logistic regression in Epi Info version 7. We calculated and report odds ratios (OR) and 95% confidence intervals (CI) and set statistical significance at p-value < 0.05. We developed choropleth maps using QGIS version 3.6.3 to show the regional and district distributions of neonatal sepsis grouped into four categories. The category ‘0.0’ represents districts that either did not report any case or those that had missing data. The category ‘0.1–17.4’ represents districts that had incidences less than or equal to the total average incidence of 17.4/1,000 LB found in this study. The category ‘17.5–46.9’ represents districts with incidences greater than the total average of 17.4/1,000 LB up to the low- and middle-income countries’ sepsis incidence of 46.9 per 1,000 LB [18] as the cutoff for the highest-burden districts in the map. The last category ’47.0-131.0’ represents districts with incidences greater than low- and middle-income countries’ sepsis incidence and above.

## Results

### Trends in incidence of inpatient neonatal sepsis nationally, Uganda, 2016–2020

Nationally, a total of 95,983 cases of inpatient neonatal sepsis (EOS and LOS combined) were reported during 2016–2020. Of these, 71,262 (75%) were EOS. On average, 1.7% of all LB neonates delivered in HC III-level facilities and above during the evaluation period experienced sepsis (Table 2).

**Table 2:** Neonatal sepsis cases and total live births, Uganda, 2016–2020

Year	LB†	Total sepsis	% among LB*	EOS^π^	% among LB^¶^	LOS^β^	% among LB^§^
2016	959,078	16,717	1.7	11,249	1.2	5,468	0.6
2017	1,040,265	18,096	1.7	13,301	1.3	4,795	0.5
2018	1,123,279	19,328	1.7	14,838	1.3	4,490	0.4
2019	1,176,931	20,515	1.7	15,674	1.3	4,841	0.4
2020	1,205,995	21,327	1.8	16,200	1.3	5,127	0.4
OVERALL	5,505,548	95,983	1.7	71,262	1.3	24,721	0.4

†LB- Live births delivered in HC III-level facilities and above. *Proportion of total sepsis among live births. ^π^EOS- Early-onset sepsis. ^¶^Proportion of EOS among live births. ^β^LOS- Late-onset sepsis. ^§^Proportion of LOS among live births

**Fig. 1:**
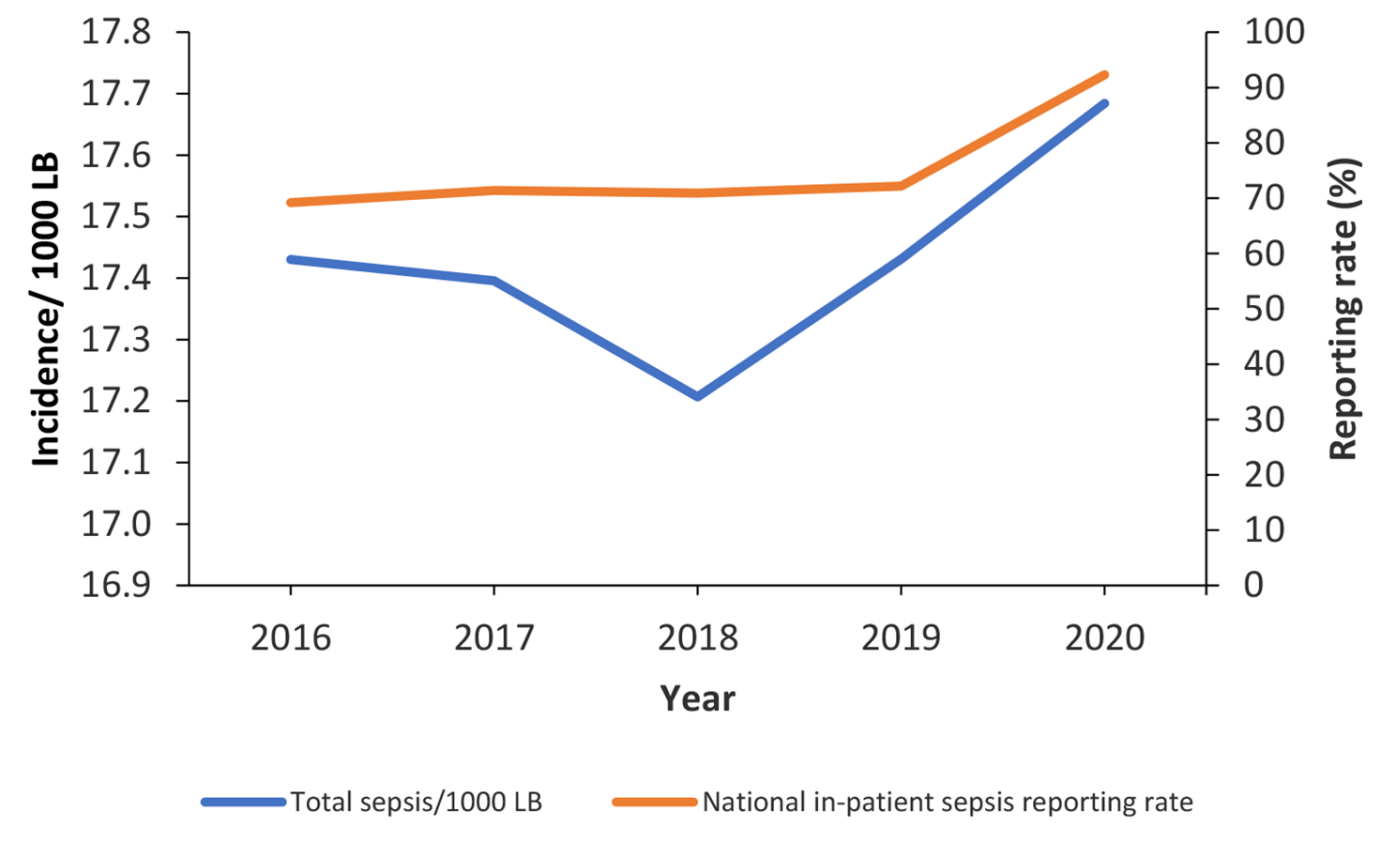
Trends in incidence of total neonatal sepsis and facility reporting rate (proportion of facilities reporting), Uganda, 2016–2020

Overall incidence of total sepsis increased slightly but non-significantly from 2016 to 2020 (p = 0.13) (Fig. 1). There was also an increasing reporting rate for inpatient neonatal sepsis data during 2016–2020 (69–92%) (Fig. 1).

**Fig. 2:**
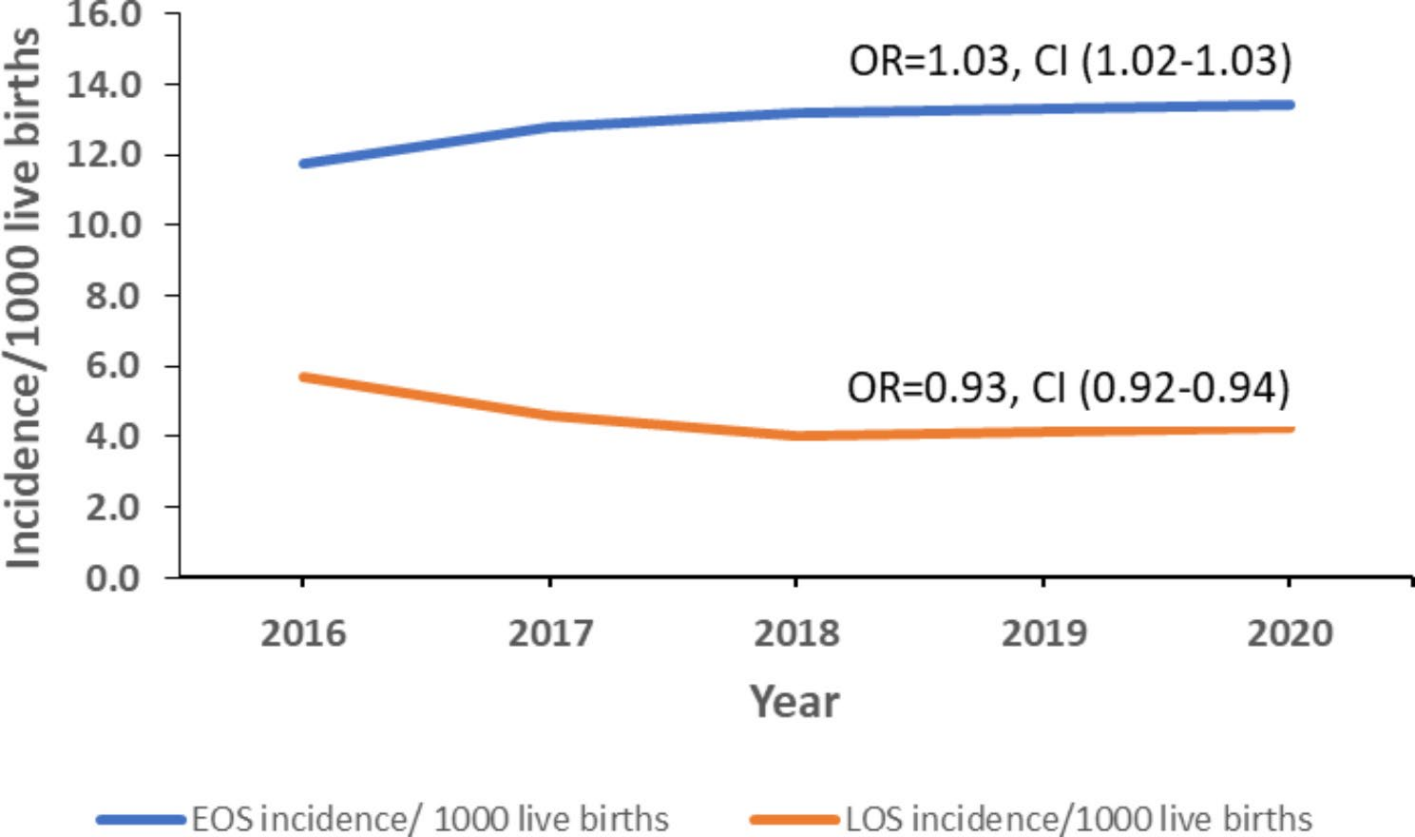
Trends in incidence of early-onset and late-onset neonatal sepsis, Uganda, 2016–2020

During 2016–2020, EOS incidence increased from 11.7 to 13.4 cases/1,000 LB, with an average yearly increase of 3% (OR: 1.03, CI: 1.02–1.03, p < 0.0005). Late-onset sepsis declined from 5.7 to 4.3 cases/1,000 LB with an average yearly decrease of 7% (OR: 0.93, CI: 0.92–0.94, p < 0.001) (Fig. 2).

## Trends in incidence of inpatient neonatal sepsis at regional level, Uganda, 2016–2020

At the regional level, EOS as well as total sepsis incidence increased in the Central and Northern regions but decreased in Western and Eastern Uganda. However, LOS only increased in Central Region during the evaluation period while other regions registered decreases. These changes were statistically significant (Table 3).

**Table 3:** Changes in neonatal sepsis incidence rates at regional level, Uganda, 2016 and 2020

Variable	IR^§ 2^016	IR 2020	OR	95% CI	P-value
Total sepsis					
Western	23.7	17.0	0.7	0.7–0.7	< 0.001
Central	15.5	23.0	1.5	1.4–1.6	< 0.001
Northern	15.3	22.2	1.2	1.2–1.3	< 0.001
Eastern	15.0	8.9	0.6	0.6–0.6	< 0.001
Early-onset sepsis					
Western	15.4	14.1	0.9	0.9–0.9	< 0.001
Central	11.8	16	1.4	1.3–1.4	< 0.001
Northern	8.3	16.8	2.0	1.9–2.2	< 0.001
Eastern	10.9	7.3	0.7	0.6–0.7	< 0.001
Late-onset sepsis					
Western	8.3	2.9	0.3	0.3–0.4	< 0.001
Central	3.7	7.0	1.9	1.8-2.0	< 0.001
Northern	7.0	5.5	0.8	0.7–0.8	< 0.001
Eastern	4.1	1.7	0.4	0.4–0.5	< 0.001

^§^IR = Incidence rate

### Spatial distribution of neonatal sepsis incidence rates at district level, Uganda, 2016–2020

**Fig. 3:**
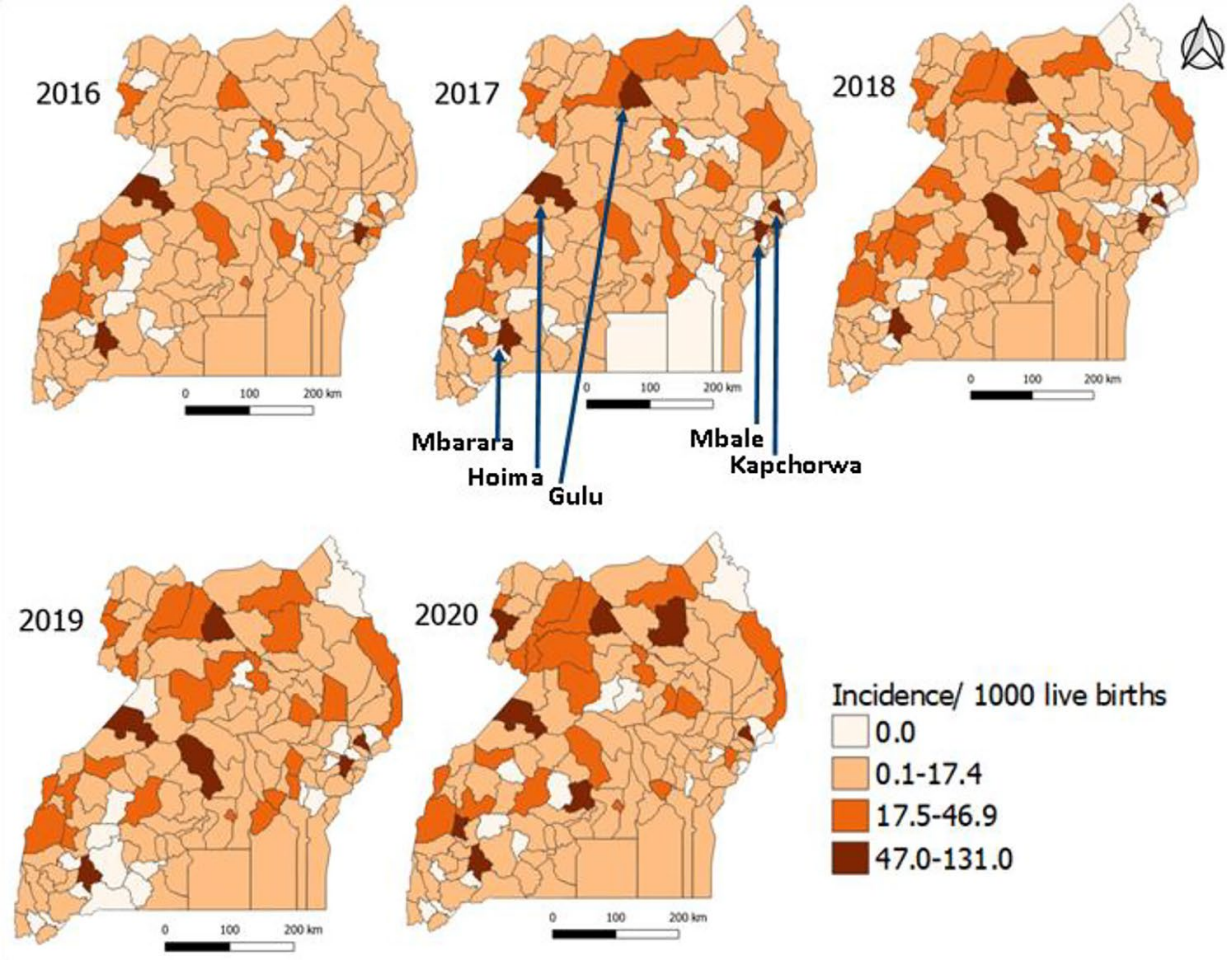
Spatial distribution of total neonatal sepsis incidence rates, Uganda, 2016–2020

We observed higher neonatal sepsis incidences among districts in Western and Northern Uganda compared to those in other regions, although incidence declined in Western Region while there was an increase in the Northern Region. Generally, an increasing number of districts showed incidence rates in the highest category (≥ 47.0/1,000 LB) from 2016 to 2020. The highest number of districts [8] with incidences > 47.0 cases/1,000 LB occurred in 2020, and included three districts (Gulu, Agago, Arua) from Northern Region, three from Western Region (Mbarara, Hoima, Kitagwenda), one from Central Region (Mityana), and one from Eastern Region (Kapchorwa) (Fig. 3).

### Incidence rate of inpatient neonatal sepsis at health facility level, Uganda, 2016–2020

**Fig. 4:**
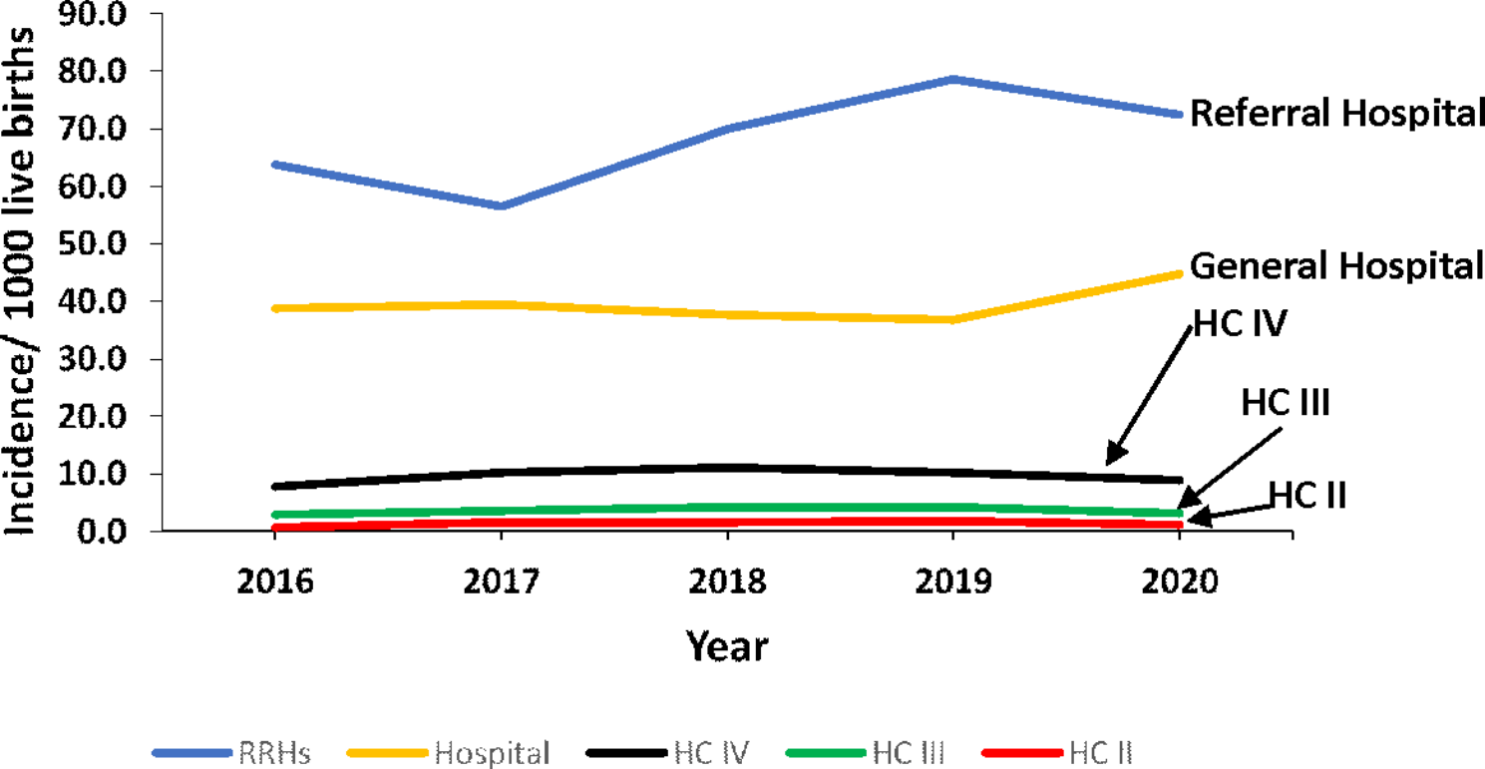
Total neonatal sepsis incidence rate by health facility level, Uganda, 2016–2020

Incidence of sepsis was highest in regional and national referral hospitals (the highest levels of health care) and decreased with each successive decreasing level of health care (Fig. 4).

## Discussion

Public health interventions to reduce neonatal morbidity and mortality, including those aimed at reducing neonatal sepsis, require monitoring to evaluate their impact. We found a neonatal sepsis incidence rate of 17.4/1,000 LB in Ugandan healthcare facilities (1.7% of all LB) during 2016–2020. Three-quarters of these cases were EOS. While LOS rates declined nationally over the study period, EOS rates increased. The increase in reporting rate of 

neonatal sepsis data likely contributed to the increase in incidence. Regional and national hospitals had higher sepsis incidence compared with lower-level health facilities.

The finding of predominantly EOS in this study is similar to the 2.6-fold higher incidence of EOS than LOS that was reported globally [14] and to the 80% EOS proportion among all sepsis that was found in a hospital-based study in southwestern Uganda [29]. In contrast to our findings, a study in India revealed that the ratio of LOS to EOS cases from 1998 to 2019 was approximately 3:1 [30]. However, the study in India utilized community-based data, which are more likely to include higher proportions of LOS, compared to facility-based data.

Regional and national hospitals had higher sepsis incidence compared with lower-level health facilities. This could potentially be due to the specialised care available at higher-level facilities, which may encourage people with serious conditions to seek care at these facilities first, or be referred there. Nalwadda et al. [31] reported that the availability of newborn sepsis drugs at HC IIs (8%) was much lower than it was for HC IIIs (67%), or HC IVs and hospitals (75%).

The decline in LOS rates from 2016 to 2020 suggests improvements in newborn care in the neonatal period in Uganda. Uganda national demographic data show increasing rates of healthcare worker checks (by a doctor, nurse, midwife, CHW, or traditional birth attendant) within two days of birth from 33% to 2011 [32] to 54% in 2016 [11]. Increased coverage of postnatal visits could contribute to decreased LOS as it provides opportunities for health education on infection prevention and control for mothers and newborn caregivers.

Although overall rates of EOS increased in the Central and Northern regions, they decreased in the Western and Eastern regions. LOS increased in the Central Region but decreased in the three other regions. Incidences of both EOS and LOS could have increased in the Central Region because of a combination of having all the NRHs [25] and being the most highly populated region [24]. NRHs have the highest level of specialized care and handle complicated obstetric and neonatal cases from all regions. A bigger population in Central Region represents a higher patient population with its resultant effects including overcrowding at health facilities, stockouts of drugs, and staff shortage which are likely risks for neonatal sepsis in constrained health systems.

In Nigeria, LOS was associated with delivery outside of a health facility [33]. In Uganda, a decline in home deliveries from 42% in 2011 [32] to 26% in 2016 [11] was accompanied by an increase in health facility deliveries from 57% in 2011 to 73% in 2016 [11]. These may have contributed to declines in LOS seen in three of Uganda’s four regions. Beyond this, several interventions and studies in the Eastern region may have had persistent impact on neonatal sepsis. The Uganda Newborn Study (UNEST) was conducted in two districts of Eastern Region in 2011, and evaluated a home visit package by CHWs alongside health facility strengthening [34]. UNEST aimed to provide context-specific information on strategies for improved newborn care following a joint WHO and UNICEF recommendation of home visits for improved newborn survival in high-mortality settings [35]. This study led to increases in skilled attendance at births. Place of delivery notwithstanding, home visits from CHWs improved newborn care practices in Eastern Region, including dry umbilical cord care and identification of danger signs of illness [34]. Further evidence in Uganda, Malawi and South Asia shows that the use of CHWs to deliver care to mothers and newborns has the potential to prevent neonatal sepsis [30, 36] and promote newborn care practices [37, 38]. Since EOS usually results from infection acquired in utero or during the birth process, the periods preceding and immediately after birth present important preventive periods. Thus, expanding the use of CHW practices in Uganda may facilitate reductions in EOS as well. Guidelines to improve essential maternal and newborn care were launched in October 2021 by the Ministry of Health. These guidelines highlight general management of newborns that reduces development of sepsis and stipulate recommended treatment [17]. Widespread rollout and implementation of these guidelines by the Ministry of Health might improve maternal and neonatal outcomes. Routine care for all pregnant women and neonates includes ensuring a clean delivery environment [39]. Health workers should continuously strive to create hygienic birthing environments. Harnessing antenatal care visits as points for education of pregnant women on neonatal sepsis prevention strategies may facilitate reductions in sepsis.

Our study had limitations. First, we were unable to identify duplicate records for neonatal sepsis cases that may have been referred from one facility to another. Second, the cases reported in DHIS2 are only those that make it to the health facilities. This almost certainly led to an underestimation of the overall burden of neonatal sepsis due to healthcare access bias. This underestimation would particularly affect estimates of LOS. In addition, the reporting rate for the study increased over time from 69% in 2016 to 92% by 2020. This likely led to underestimates of total cases in the early years of the study period; however, since we were comparing rates, it may or may not have affected the interpretation of results. Neonatal sepsis diagnosis in resource limited settings like Uganda does not have to be laboratory confirmed. This could have led to overdiagnosis.

The increase in health facility deliveries in Uganda over the years could have contributed to the increased detection and incidence of neonatal sepsis. Additionally, we were unable to ascertain the proportion of deliveries occurring at home due to incomplete reporting of home births in the DHIS2.

## Conclusion

Despite advances in health care, neonatal sepsis especially in the first seven days of life remains a challenge in Uganda. Scaling up preventive measures in the most affected settings, including in Central and Northern Uganda, the most affected districts, and at regional and national referral hospitals, could potentially reduce the burden of neonatal sepsis. Mothers should be encouraged to identify signs of infection they may be harbouring especially towards term pregnancy so that they seek treatment early. Home visits for essential newborn care by CHWs should be scaled up and widely implemented as their utility has been demonstrated in settings with substantial proportions of home births.

## Data Availability

The data upon which our findings are based belongs to the Uganda Ministry of Health and cannot be shared publicly. However, it can be made available by the corresponding author with permission from the Ministry of Health Uganda, Division of Health Information and Uganda Public Health Fellowship Program.

## Abbreviations

DHIS2: District Health Information System version 2
EOS: Early-onset sepsis
FY: Financial year
HMIS: Health Management Information System
LB: Live-birth
LOS: Late-onset sepsis
MPDSR: Maternal Perinatal Death Surveillance and Response
NMR: Neonatal Mortality Rate
PFP: Private for Profit
PNFP: Private Not for Profit
RRH: Regional Referral Hospital
